# Efficacy of Jiangzhi Mai’an Granules in the treatment of stable angina pectoris with phlegm and blood stasis obstruction and carotid artery plaque: A prospective randomized study

**DOI:** 10.1097/MD.0000000000040787

**Published:** 2024-12-06

**Authors:** Jianhua Fan, Chang Yu, Huan Tang, Zhuang Zhang, Qiong Wu, Licheng Lu, Cun Xu, Zhaochen Xia, Haixiang Xu, Jiasheng Wen, Wen Pan

**Affiliations:** a Department of Cardiology, Kunshan Hospital of Traditional Chinese Medicine, Suzhou, Jiangsu, China; b Department of Cardiology, Kunshan Integrated Traditional Chinese and Western Medicine Hospital, Suzhou, Jiangsu, China.

**Keywords:** carotid artery plaque, Jiangzhi Mai’an Granules, stable angia, traditional Chinese medicine

## Abstract

**Background:**

Stable angina pectoris, resulting from coronary artery atherosclerosis, significantly affects quality of life and carries a high risk of cardiovascular events. Despite modern therapies, managing this condition remains challenging. Traditional Chinese medicine (TCM) views it as a syndrome of heart meridian obstruction by phlegm and blood stasis, necessitating improved circulation and phlegm resolution. This study aims to evaluate the efficacy of Jiangzhi Mai’an Granules, a TCM formulation, in treating stable angina pectoris and carotid atherosclerosis, with the goal of integrating TCM with Western medicine to enhance clinical outcomes and patients’ quality of life.

**Methods:**

A total of 120 patients diagnosed with stable angina pectoris and carotid atherosclerotic plaques of the phlegm and blood stasis types were randomly divided into 2 groups. The control group (n = 60) received standard Western medical treatment, whereas the treated group (n = 60) received JZMA in addition to the standard regimen. The treatment duration in both the groups was 3 months. The outcomes measured included carotid intima-media thickness (CIMT), carotid plaque dimensions, TCM syndrome scores, and serum lipid profiles (total cholesterol [TC], triglycerides [TG], low-density lipoprotein cholesterol [LDL-C], high-density lipoprotein cholesterol [HDL-C], apolipoprotein B [Apo B], and non-HDL-C) and lipoprotein phospholipase A2 (Lp-PLA2) levels before and after treatment.

**Results:**

The treated group achieved a total efficacy rate of 93.3% (56/60), which surpassed the control group rate of 78.3% (47/60). The CIMT, carotid plaque thickness, and area in the treated group were significantly reduced posttreatment compared to baseline (*P *< .05) and were also lower than those in the control group (*P *< .05). Serum lipid levels, including TC, TG, LDL-C, ApoB and non-HDL-C were significantly decreased in both groups posttreatment (*P *< .05), and the treated group showed a further increase in HDL-C levels (*P *< .05). The treated group exhibited lower serum lipid levels than the control group posttreatment (*P *< .05). The TCM syndrome scores improved significantly in both groups after treatment (*P *< .05), with the treated group demonstrating a more pronounced reduction in scores than the control group (*P* < .05). The incidence of adverse events did not differ significantly between the 2 groups.

**Conclusions:**

JZMA combined with standard treatment effectively reduced CIMT, plaque size, and serum lipid levels, thereby enhancing clinical outcomes in patients with stable angina and carotid atherosclerosis.

## 1. Introduction

Stable angina pectoris, a common manifestation of coronary artery disease, is primarily characterized by chest pain due to myocardial ischemia resulting from atherosclerosis of the coronary arteries.^[1]^ This condition significantly impacts patients’ quality of life and is associated with a higher risk of cardiovascular events.

The burden of atherosclerosis is further underscored by its correlation with carotid artery plaques, which serve as surrogate markers for systemic vascular health and cardiovascular risk.^[2,3]^ Despite the advent of modern medical therapies, including antiplatelet agents, lipid-lowering medications, and revascularization procedures, the management of stable angina pectoris remains a clinical challenge, particularly in mitigating the progression of atherosclerotic plaques and improving long-term cardiovascular outcomes.

Traditional Chinese Medicine (TCM) offers a unique perspective on stable angina pectoris, viewing it as a syndrome resulting from obstruction of the heart meridians by phlegm and blood stasis. This classification emphasizes the importance of restoring blood circulation and resolving phlegm to alleviate the symptoms.^[4–6]^

Jiangzhi Mai’an Granules (JZMA) is a TCM formulation that embodies this therapeutic philosophy. Comprising a synergistic blend of herbs with established cardioprotective, lipid-lowering, and anti-inflammatory properties, JZMA has been used to treat conditions such as hyperlipidemia and fatty liver disease. However, its potential role in the management of stable angina pectoris and carotid artery plaques remains unclear.

Despite the promising therapeutic potential of JZMA, there remains a significant gap in the literature regarding its efficacy instable angina pectoris and carotid artery plaques. Previous studies have primarily focused on the isolated components of TCM or Western pharmacological agents, often neglecting the synergistic effects of combined therapies. Furthermore, while existing research has documented the benefits of JZMA in treating hyperlipidemia and related cardiovascular conditions, its specific impact on carotid artery atherosclerosis and associated symptoms in patients with stable angina has not been thoroughly investigated.

This study aimed to fill these gaps by assessing the efficacy of JZMA in treating stable angina pectoris with concomitant carotid artery plaques, particularly in patients exhibiting TCM syndrome of phlegm and blood stasis obstruction. By exploring the integration of TCM with conventional Western treatment paradigms, this research seeks to provide a comprehensive understanding of JZMA’s role in improving clinical outcomes, stabilizing atherosclerotic plaques, and enhancing patients’ quality of life. The findings from this study may not only contribute to the body of knowledge surrounding TCM but also offer novel insights into the management of stable angina pectoris.

## 2. Materials and methods

### 2.1. Ethics statement

This study was performed in accordance with the Code of Ethics of the World Medical Association (Declaration of Helsinki, revised in 2013) for experiments involving humans. This study was approved by the Ethics Committee of Kunshan Hospital of Traditional Chinese Medicine (Approval Number: SKYD2023204). Approval was obtained before the commencement of the study to ensure that all procedures were compliant with ethical standards. Written informed consent was obtained from all participants involved in the study. The consent form was designed to be clear and understandable, detailing the purpose of the study, procedures involved, potential risks and benefits, and voluntary nature of participation. It was emphasized that participants could withdraw from the study at any time without any consequences.

### 2.2. Sample size and randomization

The sample size for this study was calculated using the G Power software 3.1 (Heinrich Heine Universität Düsseldorf). The primary outcome measure was the change in carotid intima-media thickness (CIMT) after treatment. Based on previous studies and a pilot study we conducted, we estimated the standard deviation of the change in CIMT to be approximately 0.15 mm. To detect a minimum clinically meaningful difference of 0.1 mm in CIMT between the treatment and control groups with a power of 80% at a 2-sided significance level of 0.05, we calculated that a sample size of 54 patients per group would be required. To account for potential dropouts and loss to follow-up, we inflated the sample size by 10%, resulting in a target sample size of 60 patients per group.

From January 2023 to December 2023, patients who met the predefined eligibility criteria at Kunshan Hospital of Traditional Chinese Medicine were recruited and subsequently randomly assigned to either the treated group or the control group using a computer-generated random number sequence.

An independent statistician, who was not involved in recruiting or treating participants, generated the randomization schedule. This sequence was created prior to recruitment commencement and remained concealed until the moment of assignment. The randomization ratio was set at 1:1 to ensure balance across both groups.

### 2.3. Case selection

#### 2.3.1. Western medical diagnostic criteria

Stable angina pectoris was diagnosed according to the “2019 ESC Guidelines for the diagnosis and management of chronic coronary syndromes^[7]^” and was confirmed by coronary angiography. Carotid atherosclerotic plaques were diagnosed based on the presence of noncalcified plaques detected by ultrasound with localized intimal thickening of ≥ 1.2 mm in any of the common or internal carotid arteries.

#### 2.3.2. TCM syndrome differentiation criteria

TCM syndrome was identified as the phlegm and blood stasis obstruction type, following the “Guidelines for Clinical Research on New TCMs (Experimental).” Patients exhibited symptoms such as chest tightness or stabbing pain, oppressive chest discomfort, dizziness, shortness of breath, obesity with excessive phlegm, body fatigue, dull complexion, listless facial expressions, and poor appetite. The tongue and pulse findings included a dark purple tongue with petechiae and ecchymosis, swollen tongue with tooth marks, turbid and greasy tongue coating, and a slippery and slightly tense pulse.

#### 2.3.3. Inclusion criteria

Participants were included in the study if they met the diagnostic criteria for stable angina pectoris, had evidence of carotid atherosclerotic plaques as determined by ultrasonography, and fulfilled the TCM criteria for the phlegm and blood stasis obstruction syndrome. All participants provided informed consent, voluntarily decided to participate in the study, and signed an informed consent form.

#### 2.3.4. Exclusion criteria

Patients were excluded from the study if they had a history of myocardial infarction or stroke within the past 6 months, as these events could significantly alter the disease trajectory and complicate the interpretation of treatment effects.

Participants aged > 80 years were excluded to ensure that the study cohort was representative of a population likely to benefit from JZMA treatment without the confounding effects of age-related comorbidities that may influence treatment response and safety.

Individuals with allergies to any component of JZMA were excluded to prevent allergic reactions that could confound the assessment of treatment-related adverse events.

Those with a history of carotid endarterectomy or stent implantation were excluded to ensure that the study focused on patients with native plaque morphology and to avoid the influence of prior interventions on the outcomes measured.

Patients with known intolerance or hypersensitivity to statins were excluded to prevent adverse reactions that could be mistaken for effects of JZMA.

Participants with familial hypercholesterolemia were excluded to ensure that the study evaluated the effects of JZMA in patients with nonfamilial, sporadic hyperlipidemia, which is more representative of the general population.

Patients with hepatic dysfunction, defined by elevated levels of alanine aminotransferase or aspartate aminotransferase more than twice the upper normal limit, were excluded to avoid potential hepatotoxic effects that could be exacerbated by JZMA or other study medications. Similarly, patients with renal impairment, indicated by a serum creatinine level ≥256 μmol/L and creatine phosphokinase more than 3 times the upper normal limit, were excluded due to concerns about the safety and pharmacokinetics of study medications in the setting of reduced renal function.

Finally, participants who were taking other lipid-regulating drugs such as fibrates and their derivatives were excluded to maintain a standardized lipid-lowering regimen and to prevent interactions or overlapping effects that could complicate the assessment of JZMA’s impact on serum lipid profiles.

By applying these exclusion criteria, we aimed to minimize confounding variables and enhance the internal validity of the study, ensuring that the results would be more generalizable to the patient population of interest and that the safety and efficacy of JZMA could be accurately assessed.

### 2.4. Interventions

#### 2.4.1. Control group

The control group received standard Western medical treatment, including 100 mg Aspirin Enteric-coated Tablets (Bayer HealthCare LLC, National Drug Registration Number: J20171021) once daily, orally; 20 mg atorvastatin calcium tablets (Pfizer, National Drug Registration Number: H20051407) once daily, orally; and 60 mg isosorbide mononitrate sustained-release tablets (AstraZeneca Pharmaceutical Co., Ltd, National Drug Registration Number: H20030418) once daily, orally.

#### 2.4.2. Treated group

In addition to the control group, the treated group also received JZMA Granules.

JZMA are prepared by the pharmacy department of Kunshan Hospital of Traditional Chinese Medicine, with the following detailed composition per 15g packet, as per the Suzhou Pharmaceutical Preparation Code Z04000676: Astragalus 5g, Salvia miltiorrhiza 5g, Alisma 4.67g, Cassia seed 4.67g, Hawthorn 4.33g, Barley sprout 4.33g, Lotus leaf 4.33g. Each component has been carefully selected and weighed to ensure the optimal therapeutic effect based on traditional usage and pharmacological studies. The dosage was 15.0 g orally 3 times daily.

#### 2.4.3. Course of treatment

The treatment duration for both groups was 3 months.

### 2.5. Observation indicators

#### 2.5.1. Carotid ultrasound examination

Both groups underwent pre- and posttreatment carotid ultrasound examinations using an EPQ7 Intelligent Ultrasound System (Philips Company). The procedure involved the patient lying flat, with the neck exposed. CIMT was measured 1.0 cm away from the bifurcation of each carotid artery, and the greatest thickness and surface area of the carotid plaques were assessed in the longitudinal view. The largest plaque area was measured in cases with multiple plaques.

#### 2.5.2. Laboratory indicators

Blood lipid profiles [total cholesterol (TC), triglycerides (TG), low-density lipoprotein cholesterol (LDL-C), high-density lipoprotein cholesterol (HDL-C]) and apolipoprotein B (Apo B) were analyzed using an AU5800 automatic biochemical analyzer (Beckman Coulter Company). Lipoprotein phospholipase A2 (Lp-PLA2) levels were measured using M16 magnetic immunoassay (Shenzhen Libang Precision Instruments Co., Ltd.). Non-HDL-C = TC-HDL.

#### 2.5.3. TCM syndrome score

TCM symptom scores were evaluated according to the Guidelines for the Clinical Research of New Chinese Medicine.^[8]^ Symptoms such as chest tightness, stabbing pain in the breast, palpitations, dizziness, and heaviness in the limbs were scored on a scale of 0 to 6 (none, mild, moderate, and severe, respectively) before and after treatment.

#### 2.5.4. Safety observation

Routine blood, liver, and kidney function tests were conducted, and adverse events, such as nausea, liver dysfunction, diarrhea, rash, and itching during treatment, were recorded and compared between the 2 groups.

#### 2.5.5. Criteria for efficacy

Response: The frequency, intensity, and duration of pain attacks are significantly reduced, and carotid atherosclerotic plaques shrink; response: The frequency, intensity, and duration of pain attacks are significantly reduced, and carotid atherosclerotic plaques shrink; no response: No significant improvement in angina pectoris symptoms posttreatment, and no shrinkage of carotid atherosclerotic plaques. The total clinical effective rate = [(excellent response + response)/total cases] × 100%.

### 2.6. Statistical methods

Statistical analyses were conducted using SPSS version 19.0 software. Continuous variables are presented as mean ± standard deviation and were analyzed using the Student *t* test for comparison between the 2 groups. Categorical data were compared using the chi-squared test. *P* < .05 was considered to indicate statistical significance.

## 3. Results

### 3.1. Basic characteristics of the 2 groups

**Figure 1.**
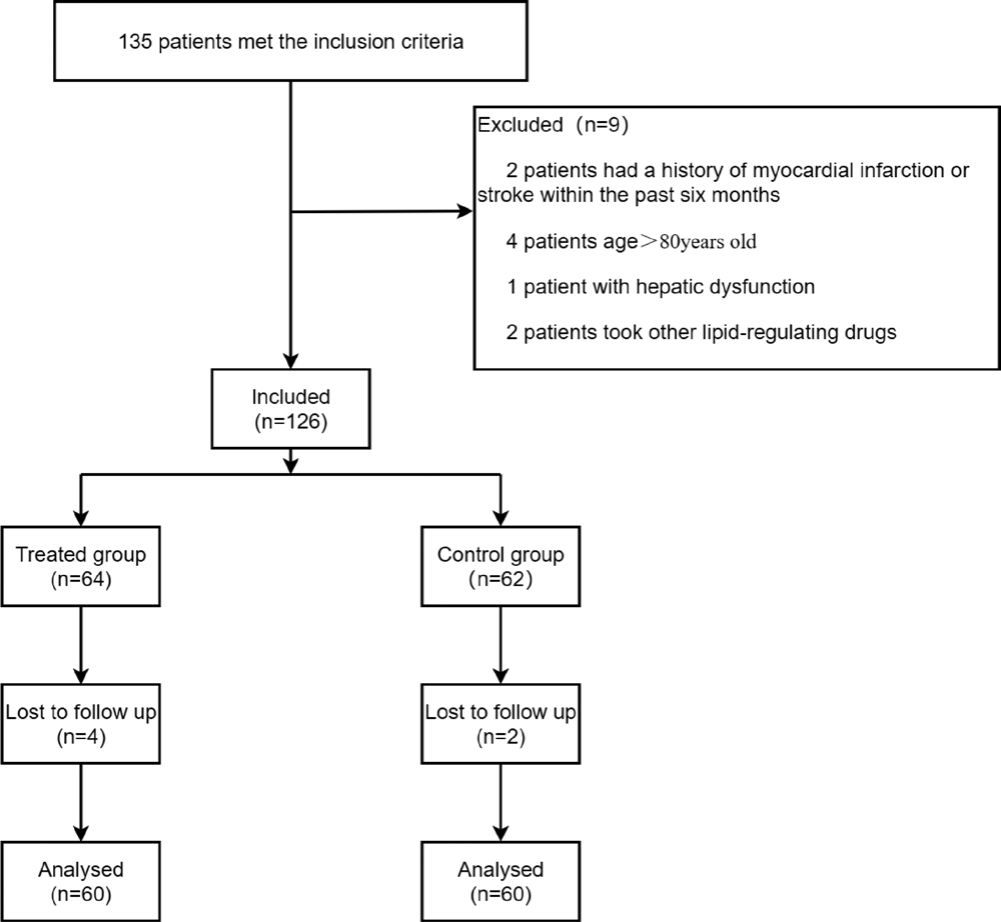
Patient enrollment diagram. This illustrates the flow of all patients screened and excluded.

During the period from January 2023 to December 2023, a total of 135 patients qualified for the study’s criteria. However, 9 patients were excluded due to certain factors. The final sample size included 126 patients. Moreover, 6 patients were also excluded due to incomplete clinical data. Ultimately, 120 patients met the inclusion criteria for this trial and were divided into 2 groups, with 60 patients in the treated group and 60 in the control group (Fig. 1).

The baseline characteristics of the participants in both the groups are presented in Table 1. No significant differences were observed between the groups with respect to demographic factors, such as sex, age, or clinical characteristics, including the prevalence of comorbidities such as hypertension, diabetes mellitus, and atrial fibrillation. Additionally, lifestyle factors, such as tobacco and alcohol consumption patterns, as well as the results of standard blood and biochemical tests, were comparable between the 2 groups.

**Table 1:** Basic characteristics of enrolled patients.

Item	Treated group (n = 60)	Control group(n = 60)	*t*	*P*
Gender (M/F, n)	38/22	34/26	0.556	.456
Age (yr)	57.15 ± 15.33	57.95 ± 14.91	0.290	.772
Hypertension [n (%)]	33 (55.0%)	30 (50%)	0.301	.583
Diabetes [n (%)]	10 (16.7%)	8 (13.3%)	0.261	.609
Atrial fibrillation [n (%)]	2 (3.3%)	5 (8.3%)	1.408	.235
Smoking [n (%)]	31 (51.7%)	32 (53.3%)	0.033	.855
Drinking [n (%)]	17 (28.3%)	23 (38.3%)	1.350	.245
White blood cell (10^9^/L)	9.24 ± 2.35	8.69 ± 2.13	−1.332	.185
Hemoglobin (g/L)	143.85 ± 15.05	139.98 ± 21.22	−1.151	.252
Platelet (10^9^/L)	225.68 ± 62.37	221.28 ± 80.73	−0.334	.739
Uric acid (µmol/L)	365.03 ± 102.63	365.45 ± 94.00	0.024	.981
Creatinine (µmol/L)	82.17 ± 33.65	83.45 ± 34.04	0.206	.837
Serum potassium (mmol/L)	4.09 ± 0.60	4.17 ± 0.65	0.711	.479
Serum sodium (mmol/L)	141.06 ± 3.87	141.00 ± 3.18	−0.085	.932
Serum chlorine (mmol/L)	102.73 ± 3.27	102.90 ± 3.25	0.291	.771

### 3.2. Effect of JZMA on carotid artery plaque

Prior to treatment, no significant differences were observed in CIMT, plaque thickness, or plaque area between the treated and control groups (*P* > .05). Following treatment, a reduction in CIMT, plaque thickness, and plaque area was noted in both the treated and control groups, with the differences being statistically significant compared to the pretreatment values (*P *< .05). Notably, the treated group exhibited significantly lower CIMT, plaque thickness, and plaque area posttreatment than the control group (*P *< .05) (Table 2).

**Table 2:** Effect of JZMA on carotid artery plaque.

Item	Control group (n = 60)		Treated group (n = 60)	
	Pre-therapy	Post-therapy	Pre-therapy	Post-therapy
CIMT (mm)	1.42 ± 0.03	1.25 ± 0.03*	1.42 ± 0.04	1.07 ± 0.03†,‡
Carotid artery plaque thickness (mm)	8.34 ± 1.29	7.00 ± 1.09*	8.17 ± 1.20	5.49 ± 0.81†,‡
Carotid artery plaque area (mm^2^)	18.54 ± 0.59	15.20 ± 0.48*	18.50 ± 0.58	10.00 ± 0.32†,‡

Pre-therapy: before corresponding treatments; post-therapy: after corresponding treatments.

Abbreviation: CIMT = carotid intima-media thickness.

* *P* < .05 relative to the control pre-therapy.

† *P* < .05 relative to the treated pre-therapy.

‡ *P *< .05 relative to the control post-therapy.

### 3.3. Effect of JZMA on blood lipids and Lp-PLA2 levels

Initially, no significant differences were noted in the levels of TC, TG, LDL-C, HDL-C, Apo B, non-HDL-C, and Lp-PLA2 between the treated and control groups (*P *> .05). Following the intervention, a statistically significant reduction in TC, TG, LDL-C, Apo B, non-HDL-C, and Lp-PLA2 levels was observed in both groups, while HDL-C levels significantly increased compared to baseline measurements (*P* < .05). Importantly, the treated group demonstrated significantly lower levels of TC, TG, LDL-C, Apo B, non-HDL-C, and Lp-PLA2, and a greater increase in HDL-C levels posttreatment compared to the control group (*P* < .05) (Table 3).

**Table 3:** Effect of JZMA on blood lipids and Lp-PLA2 levels.

Item	Control group (n = 60)		Treated group (n = 60)	
	Pre-therapy	Post-therapy	Pre-therapy	Post-therapy
TG (mmol/L)	1.51 ± 0.81	1.21 ± 0.65*	1.49 ± 0.86	0.90 ± 0.52†,‡
TC (mmol/L)	4.46 ± 1.27	3.34 ± 0.95*	4.59 ± 1.14	2.85 ± 0.70†,‡
LDL-C (mmol/L)	3.01 ± 1.13	2.61 ± 0.95*	3.15 ± 1.02	2.04 ± 0.69†,‡
HDL-C (mmol/L)	1.19 ± 0.33	1.22 ± 0.35*	1.21 ± 0.38	1.44 ± 0.45†,‡
Apo B (g/L)	1.21 ± 0.03	1.00 ± 0.02*	1.21 ± 0.03	0.86 ± 0.02†,‡
Non-HDL-C (mmol/L)	3.27 ± 1.21	2.12 ± 0.91*	3.38 ± 1.15	1.40 ± 0.78†,‡
Lp-PLA2 (ng/mL)	106.39 ± 16.13	74.47 ± 11.29*	109.33 ± 11.43	61.22 ± 6.40†,‡

Abbreviations: Apo B = apolipoprotein B, HDL-C = high-density lipoprotein cholesterol, LDL-C = low-density lipoprotein cholesterol, Lp-PLA2 = lipoprotein-related phospholipase A2, TC = total cholesterol, TG = triglycerides.

Pre-therapy: before corresponding treatments; post-therapy: after corresponding treatments.

* *P* < .05 relative to the control pre-therapy.

† *P* < .05 relative to the treated pre-therapy.

‡ *P *< .05 relative to the control post-therapy.

### 3.4. Comparison of TCM syndrome scores between the 2 groups before and after treatment

As illustrated in Table 4, a statistically significant reduction in the scores of all TCM syndromes was observed in both groups following treatment compared to the baseline scores (*P* < .05). Furthermore, the treated group exhibited significantly lower scores for all TCM syndromes posttreatment than the control group, indicating a more pronounced therapeutic effect (*P* < .05).

**Table 4:** Group comparison of TCM syndrome scores before and after treatment.

Item	Control group (n = 60)		Treated group (n = 60)	
	Pre-therapy	Post-therapy	Pre-therapy	Post-therapy
Chest oppression	4.20 ± 1.26	2.87 ± 1.44*	4.37 ± 1.35	2.10 ± 1.19†,‡
Stabbing pain in the point shanzhong (RN17)	4.17 ± 1.49	2.80 ± 1.74*	4.30 ± 1.37	2.17 ± 1.34†,‡
Palpitations and fearful throbbing	2.20 ± 1.41	1.40 ± 1.18*	2.40 ± 1.51	0.93 ± 1.30†,‡
Heavy head and clouded vision	3.67 ± 1.28	2.13 ± 1.37*	3.53 ± 1.58	1.53 ± 1.54†,‡
Fixed heavy limbs	3.57 ± 1.43	2.07 ± 1.38*	3.70 ± 1.37	1.57 ± 1.17†,‡

Notes: pre-therapy: before corresponding treatments; post-therapy: after corresponding treatments.

* *P* < .05 relative to the control pre-therapy;

† *P* < .05 relative to the treated pre-therapy;

‡ *P *< .05 relative to the control post-therapy.

### 3.5. Comparison of efficacy and adverse reactions between the 2 groups

Table 5 demonstrates a statistically significant difference in the total effective rate between the 2 groups (*P *< .05), with the treated group exhibiting superior efficacy compared with the control group. In accordance with the Guideline for the Clinical Research of New Chinese Medicine, the control group achieved a total clinical efficiency rate of 78.3%, comprising 26 patients with an excellent response, 21 with a response, and 13 with no response. In contrast, the treated group had a significantly higher total clinical efficiency rate of 93.3%, with 31 patients showing an excellent response, 25 with a response, and only 4 with no response. During the treatment period, adverse reaction events were reported in 3 patients of the control group, including liver dysfunction and general weakness. In the treated group, 2 patients reported adverse reactions (1 with liver dysfunction and 1 with a rash). Liver function abnormalities were observed in 3 cases within the control group and 2 cases in the treated group, which were presumed to be related to the intake of oral statin medications.

**Table 5:** Comparison of efficacy and adverse reactions between the 2 groups.

Item	Control group (n = 60)	Treated group (n = 60)
Total clinical effective rate (%)	47 (78.3%)	56 (93.3%)*
Excellent response	26	31
Response	21	25
no response	13	4
Adverse reactions	3	2
Liver dysfunction	2	1
Weakness	1	0
Rash	0	1

* *P* < .05 relative to the control group.

## 4. Discussion

Stable angina pectoris is a clinical syndrome characterized by acute, transient myocardial ischemia and hypoxia caused by an increased myocardial workload, underlying fixed severe stenosis of the coronary arteries, and is closely related to atherosclerosis.^[1]^Evidence suggests that carotid artery plaque lesions can serve as an indicator of coronary artery disease to a certain degree. The examination of carotid artery plaques, being simple, noninvasive, and repeatable, has gained widespread clinical utility.^[9,10]^ In Western medicine, the current therapeutic approach to stable angina pectoris primarily includes oral medications, such as statins for plaque stabilization, aspirin for antiplatelet aggregation, and nitrates for vasodilation, which have demonstrated tangible clinical benefits.^[11]^

In TCM, stable angina pectoris is classified under syndromes as chest oppression, heart oppression, or true heart pain. The etiology is predominantly attributed to the obstruction of the heart meridian by phlegm and blood stasis, causing pain when the flow is impeded. The therapeutic principle emphasizes the activation of blood circulation to disperse stasis and the resolution of phlegm to clear the meridians.

JZMA is an proprietary TCM formulation developed by our hospital, which has shown promising results in managing hyperlipidemia and chest pain. The composition of the formulation included the following key ingredients: Astragalus is known for its immunomodulatory effects, enhancing the body’s immune function and improving the antioxidant capacity of myocardial cells. It protects the heart from ischemic injury, improves microcirculation, and has hypotensive effects, reducing myocardial oxygen consumption. Salvia miltiorrhiza contains active components such as tanshinones, which promote blood circulation and remove blood stasis. It dilates the coronary arteries, increases coronary blood flow, improves myocardial ischemia, reduces blood viscosity, and prevents thrombosis through antiplatelet aggregation. Alisma has diuretic and heat-clearing properties. In cardiovascular diseases, it helps lower blood lipid levels, slows the progression of atherosclerosis, and reduces blood pressure through its diuretic effects. Cassia seed contains anthraquinone compounds and has laxative, hypotensive, and lipid-lowering effects. It also protects vascular endothelial cells through antioxidant and anti-inflammatory actions, slowing the progression of atherosclerosis. Hawthorn is rich in flavonoids and can improve cardiovascular function by enhancing myocardial contractility, reducing blood pressure, combating arrhythmias, and regulating blood lipids. Barley sprout contains various digestive enzymes and vitamins, promoting digestion and nutrient absorption. Its antioxidant properties in cardiovascular diseases help reduce oxidative stress and protect endothelial function. Lotus leaf contains flavonoids and alkaloids, offering heat-clearing and diuretic effects. It also helps lower blood lipids and improve endothelial function, contributing to the prevention of atherosclerosis. The combined actions of these herbs in JZMA improve stable angina pectoris and carotid artery plaques by enhancing rheological blood properties, reducing lipid levels, and mitigating atherosclerotic progression through antioxidant, anti-inflammatory, and endothelial protective effects.

CIMT is a well-established surrogate marker for atherosclerotic burden and a predictor of cardiovascular events.^[12,13]^ In our study, we observed a significant reduction in CIMT in the treated group after JZMA treatment compared to the control group. This reduction in the CIMT suggests that JZMA may have a stabilizing effect on atherosclerotic plaques, potentially reducing the risk of plaque rupture and subsequent cardiovascular events. Carotid plaque dimensions, including thickness and area, were also significantly reduced in the posttreatment group. This reduction indicates a potential regression of atherosclerotic plaques with JZMA treatment, which is a desirable outcome in managing patients with carotid artery disease. Dyslipidemia is a key factor in the development and progression of atherosclerosis. Our study demonstrated that JZMA treatment led to significant reductions in TC, TG, and LDL-C levels, and an increase in HDL-C levels in the treated group compared to the control group. These changes in serum lipid profiles suggest that JZMA may have a favorable impact on lipid metabolism, which could contribute to the stabilization and regression of atherosclerotic plaques.

Lp-PLA2 is an inflammatory biomarker associated with coronary artery disease.^[14]^ Previous research^[15]^ has indicated that approximately 25% of the Asian population experiences a decrease or loss of Lp-PLA2 activity. This genetic characteristic is significant, especially within the context of our study’s demographic, as it suggests that activity assays may not accurately reflect the pathological role of Lp-PLA2 in this population subset. Consequently, we opted to measure Lp-PLA2 concentration, which we believed would provide more consistent and reliable data in our study cohort. We found that JZMA treatment significantly reduced the Lp-PLA2 levels in the treated group. This reduction in Lp-PLA2 levels may indicate an anti-inflammatory effect of JZMA, which could be beneficial in reducing the inflammatory component of atherosclerotic plaques.

TCM syndrome scores, including chest oppression, stabbing pain in the chest, palpitations, and other related symptoms, were significantly reduced in the treated group after JZMA treatment. This improvement in TCM syndrome scores reflects the overall clinical benefit of JZMA in alleviating symptoms associated with stable angina pectoris and carotid artery plaques.

Regarding safety, the control group reported 2 instances of liver function abnormalities and 1 case of asthenia, whereas the treated group reported 1 case of liver function abnormality and 1 of rash. This indicates that integrating Western medicine with TCM may mitigate adverse effects while augmenting therapeutic benefits.

The findings of our study suggest that JZMA, when combined with standard treatment, can effectively reduce CIMT, plaque size, and serum lipid levels and enhance clinical outcomes in patients with stable angina and carotid atherosclerosis. These results have several important implications for clinical practice.

Firstly, the integration of the JZMA into the standard care of patients with stable angina pectoris and carotid artery plaques could potentially offer a novel therapeutic strategy to improve cardiovascular outcomes. By reducing CIMT and plaque size, JZMA may help stabilize vulnerable plaques and decrease the risk of plaque rupture, which is a key event in the development of acute coronary syndromes. This could potentially lead to a reduction in cardiovascular events and improve long-term prognosis in these patients.

Secondly, the observed improvements in serum lipid profiles following JZMA treatment suggest that it may have a favorable impact on lipid metabolism, which is a key factor in the development and progression of atherosclerosis. By reducing TC, TG, LDL-C, Apo B, and non-HDL-C levels, and increasing HDL-C levels, JZMA may contribute to the stabilization and regression of atherosclerotic plaques.

Thirdly, the significant reduction in TCM syndrome scores with JZMA treatment indicates that it can effectively alleviate symptoms associated with stable angina pectoris and carotid artery plaques, such as chest oppression, stabbing pain in the chest, and palpitations. This suggests that the JZMA could provide a valuable adjunct to standard care for improving symptom control and quality of life in these patients.

Although our study provides valuable insights into the efficacy of JZMA in treating stable angina pectoris accompanied by carotid artery plaque under the TCM pattern of phlegm and blood stasis obstruction, it is essential to acknowledge several limitations that might impact the interpretation of our results. Firstly, potential biases, such as selection bias, cannot be entirely ruled out despite our efforts to recruit participants through strict inclusion criteria. Although we aimed for a randomized controlled trial design, the recruitment process was conducted within a single center, which might introduce some inherent biases. Future studies should consider multicenter designs to mitigate this limitation. Secondly, concerning generalizability, our findings may not be directly applicable to populations outside China due to differences in genetics, lifestyle factors, and healthcare practices. Moreover, the specific TCM syndrome diagnosis used in our study was based on TCM principles, which might not be universally recognized in Western medical systems. Therefore, further international trials are warranted to validate the effectiveness of JZMA across diverse patient populations. Finally, regarding the study design, although we employed a prospective randomized approach, there is room for improvement in future research. Specifically, implementing a double-blind placebo-controlled design would provide stronger evidence of treatment efficacy. Additionally, extending the follow-up period beyond the current duration could offer deeper insights into the long-term effects of JZMA on stable angina pectoris symptoms and carotid artery plaque progression.

In conclusion, our study suggest that JZMA has the potential to become an important component of standard care for patients with stable angina pectoris and carotid artery plaques. By improving cardiovascular outcomes, lipid profiles, and symptom control, JZMA could provide valuable addition to the current treatment options available for these patients. However, further research and educational efforts are needed to fully realize the clinical implications of our findings and to guide the appropriate integration of the JZMA into standard care.

## Acknowledgments

The authors convey their deepest gratitude to all the participants of this study, whose contribution were invaluable. Additionally, they extend their thanks to the personnel at the cardiology department, acknowledging their keen interest and dedication to this research.

## Author contributions

**Conceptualization:** Wen Pan.

**Data curation:** Jianhua Fan, Chang Yu, Cun Xu, Zhaochen Xia.

**Formal analysis:** Qiong Wu, Licheng Lu.

**Supervision:** Huan Tang, Zhuang Zhang.

**Validation:** Jiasheng Wen.

**Writing – original draft:** Jianhua Fan, Chang Yu.

**Writing – review & editing:** Haixiang Xu, Wen Pan.
